# Quality of life assessment instruments for radiodermatitis in cancer patients: scoping review

**DOI:** 10.1590/1518-8345.7739.4698

**Published:** 2025-11-03

**Authors:** Letícia Moraes Lopes, Beatriz Nappo Neiva, Beatriz Regina Lima de Aguiar, Paloma Gomes, Elaine Barros Ferreira, Paula Elaine Diniz dos Reis

**Affiliations:** 1 Universidade de Brasília, Faculdade de Ciências da Saúde, Brasília, DF, Brazil; 2 Scholarship holder at the Fundação de Apoio à Pesquisa do Distrito Federal (FAPDF), Brazil; 3 Scholarship holder at the Coordenação de Aperfeiçoamento de Pessoal de Nível Superior (CAPES), Brazil; 4 Scholarship holder at the Conselho Nacional de Desenvolvimento Científico e Tecnológico (CNPq), Brazil

**Keywords:** Quality of Life, Skin, Radiodermatitis, Radiotherapy, Neoplasms, Review

## Abstract

to map the literature on skin-related quality of life questionnaires and assessment tools applied to cancer patients undergoing radiotherapy who developed radiodermatitis.

scoping review conducted in accordance with JBI recommendations. The search was conducted in seven sources of information and in the grey literature. There were no restrictions on the publication period or language of the articles. A qualitative synthesis of the data was performed, presented descriptively and through tables and figures.

thirty-two studies were included, most of which were from North America (n=14). The instruments used to assess skin-related quality of life were: the Dermatology Life Quality Index (n=17), Skindex-16 (n=11), Skindex-29 (n=1), and Padua Skin-Related Quality of Life (n=1). The most frequently applied instruments were the Dermatology Life Quality Index and Skindex-16, used predominantly in patients with breast cancer.

different instruments used to assess skin-related quality of life in patients with radiodermatitis were identified, with emphasis on Skindex-16 and DLQI. The diversity of tools points to the absence of consolidated guidelines on the choice of the best instrument to be implemented in this clinical context.

## Highlights

**(1)** DLQI, Skindex-16, Skindex-19, and PRSQ are specific instruments for radiodermatitis. **(2)** Skindex-16 assesses mental and emotional aspects more efficiently than DLQI. **(3)** The choice of instrument should consider the specificities and needs of the skin.

## Introduction

Radiodermatitis is an inflammatory adverse event that manifests as skin lesions resulting from exposure to ionizing radiation^(1)^, with a high incidence in the pelvis, breast, and head and neck regions^(1)^. In a study conducted in the interior of São Paulo, Brazil, with women who underwent breast irradiation, there was a 98.2% incidence of radiodermatitis^(2)^. In the acute phase, radiodermatitis is characterized by the presence of one or more manifestations, such as hyperpigmentation, erythema, dry desquamation, moist desquamation, necrosis, ulceration, and local infection^(1)^. This radiotoxicity often appears from the second week of treatment^(3)^.

The severity of tissue damage that occurs in radiodermatitis can vary according to the dose per fraction, the total dose administered, and individual susceptibility, such as age, body mass index, sun exposure, genetic factors, among others^(2,4-6)^. In addition, the greater the severity of radiodermatitis, the greater the possibility of discomfort, psychosocial impact, impairment in daily activities, and interruption of treatment, which can affect the patient’s prognosis^(7)^. All of these factors affect patients’ quality of life^(8)^.

Thus, health-related quality of life refers to the individual’s perception of their own living conditions in the face of the disease, as well as the treatment and progression of the disease^(9)^. Its assessment in patients who present some degree of radiodermatitis using instruments for its measurement allows for the individualization of care and the consideration of subjective factors, such as psychological state, social interactions, and self-esteem^(10)^. In addition, it facilitates care planning and the monitoring of signs, symptoms, and side effects, so that professionals can provide care based on the patient’s complaints^(10)^.

In this context, the use of questionnaires and scales to assess different contexts in the health field is increasingly common. Therefore, it is essential to ensure the accuracy and efficiency of these instruments to provide an adequate assessment of results and promote an effective action plan. In addition, it is essential to identify questionnaires and instruments that assess quality of life (QoL) that can be applied to the Brazilian population, specifically those of national origin or translated into Brazilian Portuguese.

The translation of instruments into Brazilian Portuguese provides adaptation to the cultural and linguistic context of the country and increases their credibility^(10-11)^. In addition, specific questionnaires that assess QoL in patients with radiodermatitis can bring greater reliability and validity to the results. These factors highlight the importance of identifying and analyzing instruments aimed at this purpose, a gap not yet explored in the literature. A comprehensive preliminary search conducted on review protocol registration platforms, such as the Open Science Framework (OSF) and PROSPERO, in addition to the PubMed database, did not identify any published or registered studies on the same topic

Thus, the objective of this study was to map the literature on skin-related QoL questionnaires and assessment instruments applied to cancer patients undergoing radiotherapy who developed radiodermatitis.

## Method

### Protocol and registration

This is a scoping review conducted in accordance with JBI recommendations^(12)^. The Preferred Reporting Items for Systematic Reviews and Meta Analyses (PRISMA) for Scoping Reviews (PRISMA-ScR) checklist was used to report this review^(13)^. The protocol for this scoping review was registered on the Open Science Framework (OSF) platform^(14)^ and is identified by the Digital Object Identifier (DOI) 10.17605/OSF.IO/UH42P. The protocol is publicly available at: https://osf.io/uh42p/.

This review aimed to answer the guiding question: “What evidence is available in the literature about the instruments used to measure skin-related QoL in adult cancer patients undergoing radiotherapy who developed radiodermatitis?” This question was formulated using the acronym PCC, where P (population) refers to adult cancer patients undergoing radiotherapy, C (concept) – questionnaires and instruments used to measure skin-related QoL in adult cancer patients undergoing radiotherapy who developed radiodermatitis C (context) – any environment, whether hospital, outpatient, or home, national or international, in which skin-related QoL was assessed during radiotherapy or up to 4 weeks after its completion.

### Eligibility criteria

Observational and experimental studies that measured skin-related QoL during radiotherapy, using validated or unvalidated instruments, in adult cancer patients undergoing radiotherapy who developed radiodermatitis were included. There were no restrictions on the publication period or language of the articles. Thus, studies from different countries were included, covering both national and international publications, conducted in various contexts.

Studies were excluded if they: (1) included a sample composed of children and adolescents (<18 years); (2) did not present a QoL assessment using instruments; (3) used instruments for QoL assessment that were not specific to the skin; (4) involved patients who did not undergo radiotherapy.

### Sources of information

To identify potentially relevant studies and descriptors, an initial search was conducted in the PubMed information source. Next, an electronic search strategy was designed specifically for PubMed via the National Institutes of Health (NIH) and subsequently adapted for each of the following information sources: Latin American and Caribbean Literature on Health Sciences (LILACS) via the Virtual Health Library (VHL), Web of Science Core Collection (WoSCC) via Clarivate; Embase via Elsevier, LIVIVO, and Cochrane Database via Cochrane Library. In addition, searches were conducted in the grey literature using Google Scholar and the ProQuest^TM^ Dissertation & Theses Citation Index (ProQuest) via Clarivate.

Terms related to the population of the acronym PCC were not used so as not to restrict the search. The first search strategy was constructed using MeSH Terms and keywords for use in the PubMed database. Then, the other sources of descriptors – Health Sciences Descriptors (DeCS) and Emtree – were consulted to adapt the search strategy for each database. Boolean operators (OR and AND) were used to combine the terms. The search that comprised the results of this review was conducted on January 25, 2024, in all sources of information and grey literature. The steps to conduct the review were carried out between January and October 2024.

Finally, the reference lists of the included studies were also examined to identify possible relevant studies that were not captured in the initial search strategy.

### Search strategy

The complete electronic search strategy used in PubMed, accessed through the National Institutes of Health (NIH) platform, was as follows: (“radiodermatitis”[MeSH Terms] OR “radiodermatitis”[All Fields] OR “radiation dermatitis”[All Fields] OR “radioepidermitis”[All Fields] OR “radiation reaction” [All fields] OR ‘radioepithelitis’[All fields] OR “acute radiation reactions”[All fields] OR “acute radiation-induced skin” [All fields] OR “radiation-induced damage”[All fields] OR “skin radiation syndrome” [All fields] OR “radiodermatitis”[All fields] OR “radiation-induced dermatitis”[All fields] OR “radiation-induced skin lesions”[All fields] OR “acute radiation-induced toxicity”[All fields] OR “radiation-induced toxicity” [All fields] OR “radiation-induced toxicities” [All fields] OR “radiation-induced toxicity in normal tissues” [All fields] OR “skin reaction” [All fields] OR “skin reactions” [All fields] OR “skin toxicity” [All fields] OR “skin toxicities” [All fields] OR “radiation-induced side effects” [All fields] OR “radiation toxicity” [All fields] OR “tissue complications” [All fields] OR “radiation injury” [All fields]) AND (“quality of life” [MeSH Terms] OR “quality of life” [All fields] OR “health-related quality of life” [All fields] OR “HRQL” [All fields] OR “HOL” [All fields] OR ‘hrqols’ [All fields] OR “quality of life” [All fields] OR “hrqol” [All fields] OR “health-related quality of life” [All fields] OR “quality of life” [All fields]) AND (“questionnaire” [All fields] OR ‘questionnaires’ [All fields] OR “surveys and questionnaires” [MeSH Terms] OR “surveys and questionnaires” [All fields] OR “questionnaire” [All fields] OR “questionnaires” [All fields] OR “measurability” [All fields] OR “measurable” [All fields] OR ‘measurably’ [All fields] OR “measurements” [All fields] OR “measurable” [All fields] OR “measured” [All fields] OR “measurement” [All fields] OR “measurements” [All fields] OR ‘measurements’ [All fields] OR “measurer” [All fields] OR “measurers” [All fields] OR “measure” [All fields] OR “measurements” [All fields] OR “measurement” [All fields] OR ‘measurements’ [All fields] OR “weights and measures” [MeSH terms] OR “instrument” [All fields] OR “instruments” [All fields] OR “instrumentation” [All fields] OR “instruments” [All fields] OR “instrumented” [All fields] OR ‘instrumentation’ [All fields] OR “surveys” [All fields]).

### Selection of evidence sources

After identifying the studies in the information sources, they were exported to the EndNoteBasic^®^ reference management software (Thomson Reuters, USA), online version, for automatic exclusion of duplicates. The references were then sent to Rayyan software, where the titles and abstracts were independently evaluated by two reviewers (L.M.L. and B.N.N.). In this process, studies that did not meet the eligibility criteria were excluded. Next, the selected references were read in full by the same reviewers, and again, those that did not meet the eligibility criteria were excluded from this review. In both stages, if there was a disagreement between the two reviewers about the inclusion of a study, a third reviewer (P.E.D.R.), with expertise in the subject, was called upon to decide on the inclusion or exclusion of the study in this review. The selected studies proceeded to the data extraction phase. Articles published between 2004 and 2023 were selected.

### Data extraction process and data collected

For data extraction, a standardized table in Microsoft Excel software was used, developed by the authors, containing information related to publications and PCC domains. Data extraction from the included studies was performed by the first reviewer (L.M.L.) and checked by the second reviewer (B.N.N.). The following data were collected: study characteristics (year of publication and country), sample characteristics (type of cancer and presence of radiodermatitis), and characteristics of health-related QoL assessment instruments applied to patients with radiodermatitis. If any of these data were not available in the study, three emails were sent at 15-day intervals requesting the information from the corresponding author.

In addition, a detailed evaluation of the instruments was included in this review, based on data collection from the original reference of the instrument, to present the following information: validation for the Portuguese language, items that comprise the instrument, scale and score for each item, method of application of the instrument, method of evaluation of the results obtained with the instrument, and average response time.

### Summary of results

The primary outcome of this scoping review was to describe the instruments used to assess skin-related QoL in cancer patients undergoing radiotherapy who developed radiodermatitis. In addition, the number of studies published per year, the distribution of the use of QoL assessment instruments by geographic region, the characteristics of the instruments that measured QoL, and the frequency of instruments used by cancer type were verified. The data were analyzed using qualitative synthesis and presented descriptively and through tables and figures.

### Ethical considerations

As this is a secondary study that uses previously published primary studies as its data source, this work does not require review by the Research Ethics Committee.

## Results

### Selection of studies

A total of 1,371 references were identified in the information sources, and after automatic removal of duplicate studies by Endnote software, 1,198 studies remained. After this step, the references were forwarded to Rayyan software, where another 207 duplicate studies were manually removed. A total of 991 references were then read for titles and abstracts. After applying the eligibility criteria, 919 studies were excluded, and 72 were read in full. Twenty-three restricted-access studies were not retrieved for full reading, even after attempting to contact the authors. The remaining 49 studies were analyzed for eligibility, resulting in 24 studies excluded and 25 included in this scoping review from the search of information sources.

From the grey literature and manual search of the reference list of the included studies obtained from the information sources, we identified 29 scientific publications, of which 7 duplicates were excluded and 13 were not retrieved. For studies not available in full, three emails were sent at 15-day intervals requesting the information from the corresponding author. All attempts were unsuccessful. Thus, nine studies proceeded to the title and abstract evaluation stage and then to full-text reading. After applying the eligibility criteria, seven studies from the search of other sources were included in the review.

**Figure 1-:**
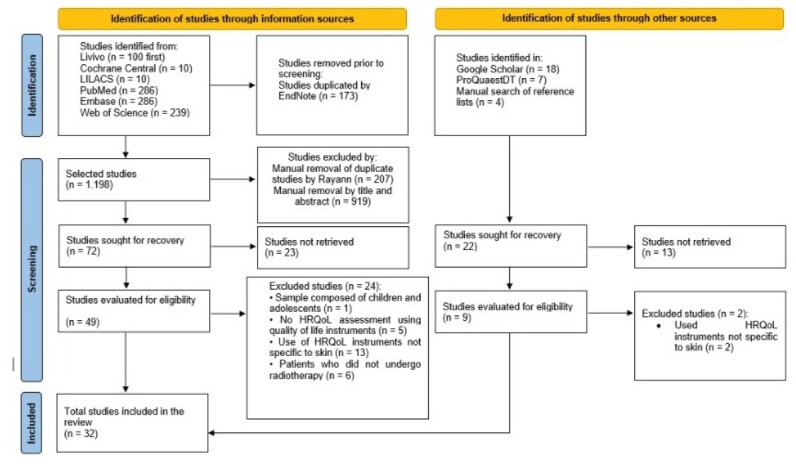
Flowchart of the process for identifying, screening, and including studies. Adapted from PRISMA (2020)^(47)^

Thus, a total of 32 studies were included in this scoping review^(15-46)^. The process of searching and selecting studies is detailed in Figure 1.

### Study characteristics

**Figure 2-:**
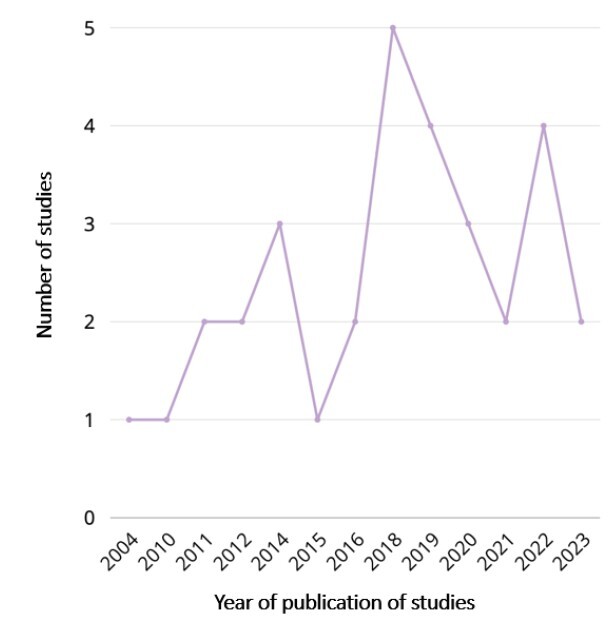
Number of studies included in this scoping review by year of publication

All included studies were published in English between 2004 and 2023, with a peak in publications in 2018 (5 studies), followed by 2019 and 2022, with 4 studies each (Figure 2).

With regard to the continent of origin of the publications, North America has the highest number of studies (n = 14; 43.75%), all conducted in the United States of America (USA)^(16-21,27,29,34,36,41-44)^. Next is Europe, with 7 publications, 4 from Belgium^(22,37-39)^, 2 from the United Kingdom^(28,46)^, and 1 from Italy^(30)^. Asia has 6 studies, 4 of which are from Iran^(31-33,35)^, 1 from Japan^(45)^, and 1 from Thailand^(25)^. Oceania has 2 studies, both from Australia^(23-24)^. South America also has 2 studies, both from Brazil^(26,40)^. Finally, Africa has 1 study, from Egypt^(15)^.

### Results of individual studies

The skin-related QoL instruments identified were: the Dermatology Life Quality Index (DLQI)^(16-20,26,28,31-33,46)^, Skindex-16^(15,22-25,27,29-30,34-36,38-39,41-45)^, Skindex-29^(37)^, and Padua Skin-Related Quality of Life (PSRQ)^(21)^. The Skindex-16 was reported in studies conducted in the USA (n=8; 25%), Belgium (n=3; 9.375%), Australia (n=2; 6.25%), Italy (n=1; 3.125%), Thailand (n=1; 3.125%), Iran (n=1; 3.125%), and Egypt (n=1; 3.125%). The DLQI was reported in studies conducted in the USA (n=5; 15.625%), Iran (n=3; 9.375%), the United Kingdom (n=2; 6.25%), and Brazil (n=1; 3.125%). The Skindex-29 and PSRQ instruments were reported in one study each, conducted in Belgium and the USA, respectively.

**Figure 3-:**
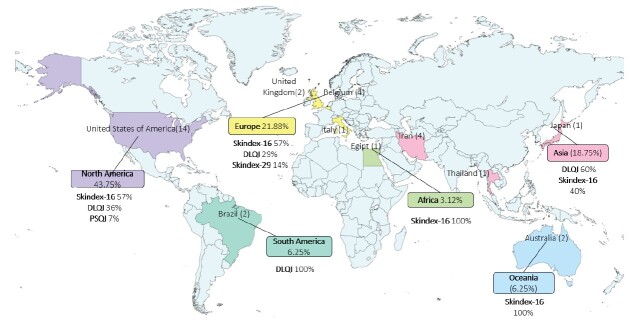
Frequency of skin-related QoL instruments in patients with radiodermatitis by continent

Thus, the use of Skindex-16 predominates in Africa, North America, Europe, and Oceania, while DLQI predominates in South America and Asia (Figure 3). The systematic reviews, one from Brazil^(40)^ and the other from Japan^(45)^, were not included in Figure 3 to avoid double counting of primary studies.

The DLQI^(48)^ instrument aims to quantify how much a skin condition affects a person’s QoL. It is a questionnaire consisting of 10 items divided into 6 categories, namely: symptoms and feelings (2 items), daily activities (2 items), leisure (2 items), work and study (1 item), interpersonal relationships (2 items), and treatment (1 item). Each item allows for a score of 0 (none) to 3 (very much), with the total score ranging from 0 to 30 points. As with other questionnaires, it is important to analyze the sociocultural and economic context of the population, as different emphases can be observed in the questions answered.

The Skindex^(49)^ is a dermatological QoL assessment tool that aims to measure the impact of dermatological conditions and their effects on individuals’ QoL. In addition, it makes it possible to distinguish between different skin manifestations—such as psoriasis, melasma, chronic ulcers, vitiligo, rosacea, among others—and monitor their evolution over a period of time. It assesses three domains: functional, emotions, and symptoms.

In this review, Skindex-16^(15,22-25,27,29-30,34-36,38-39,41-45)^ and Skindex-29^(37)^ were identified, which have specificities in common. In both questionnaires, the item is disregarded when there is more than one answer for it. For these instruments, it is possible to quantify each domain by calculating the average score of the answers using a three-scale scoring system. In other words, each domain has an average and is assessed separately from the others.

The Skindex-29^(49)^ consists of 29 items divided into the domains of emotions (10 items), symptoms (7 items), and functioning (12 items). The score is 1 point for “never” and 5 points for “all the time.” The Skindex-29 score is then transferred to a linear scale, which ranges from 0 to 100 for each domain, where the higher the score, the greater the effect on the individual’s QoL. The total score for the Quality of Life assessment can result in 0 points (never), 25 points (rarely), 50 points (sometimes), 75 points (often), and 100 points (all the time). The final score consists of the average score for each domain. The cutoff to identify patients with severely impaired QoL is ≥ 52 for the symptoms domain, ≥ 39 for the emotions domain, ≥ 37 for the functional domain, and ≥ 44 for the total score.

The Skindex-16^(49)^ has 16 items, of which 7 are for the domain of emotions, 4 for symptoms, and 5 for functionality. This instrument was developed from the Skindex-29 with the aim of being more succinct, accurate, and efficient. Its construction was based on maintaining items that performed best in the Skindex-29 and adding new questions. The scale used is Likert-type, and the scoring of its items ranges from 0 points for “never bothered” to 6 points for “always bothered”. The total score for each item is also transformed into a linear scale from 0 to 100 points, and the score for each domain of the Skindex-16 consists of the average of its items.

Finally, the PSRQ^(50)^ is an Italian questionnaire, initially designed with 63 items, to measure both the level of comfort and discomfort related to the skin. Currently, the PSRQ consists of 50 items, each of which allows a response ranging from 1 “strongly disagree” to 5 “strongly agree”. The instrument has 5 domains, namely interpersonal commitment (12 items), positive feelings and emotions (17 items), negative feelings and emotions (13 items), stress, and physical commitment (8 items). The result can be obtained by summing all items or by domain, by summing the average score obtained from the responses to each set of items that make up the domain.

For all instruments presented, the higher the score, the greater the commitment to skin-related QoL. The main characteristics of the instruments are presented in Figure 4.

**Figure 4–:** Characteristics of skin-related QoL instruments identified in the selected articles (n = 32). Brasília, DF, Brazil, 2024

Instrument	Validated for Portuguese language	Number of items	Number of categories	Category names	Score	Score assigned to the item	Response time in minutes
Dermatology Life Quality Index (DLQI) ^( 48 , 51 )^	Yes	10	6	1. Symptoms and feelings	Maximum: 30 / Minimum: 0	0 to 3 points for each item	Approximately 2
				2. Daily activities		0. Not at all	
				3. Leisure		1. A little	
				4. Work and study		2. A lot	
				5. Interpersonal relationships		3. Very much	
				6. Treatment			
Skindex-16 ^( 49 , 52 )^	Yes	16	3	1.Emotions	Maximum: 96 / Minimum: 0	0 to 6 points for each item	2-3
				2.Daily activities		0. Never bothered me	
				3.Symptoms		6. Always bothered me	
Skindex-29 ^( 49 , 53 )^	Yes	29	3	1.Emotions	Maximum: all the time/ Minimum: never	1. Never	Approximately 5
				2.Daily activities		2. Rarely	
				3.Symptoms		3. Sometimes	
						4. Often	
						5. All the time	
Padua Skin-Related QoL Questionnaire ^( 50 )^	No	50	5	1. Interpersonal commitment	Maximum: 250/ Minimum: 50	1 to 5 points for each item	-
				2. Positive feelings and emotions		1. Strongly disagree	
				3. Negative feelings and emotions		5. Strongly agree	
				4. Stress			
				5. Physical commitment			

**Figure 5-:**
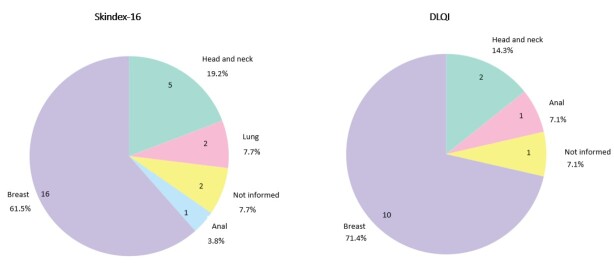
Frequency of cancer types presented by patients in studies using Skindex-16 and DLQI

Skindex-16 and DLQI were the most used instruments to assess QoL related to radiodermatitis (Figure 5). Regarding the type of cancer of the participants in the included studies, it was found that QoL was assessed mainly in individuals with breast cancer (16 studies used Skindex-16^(15,22-25,27,30,34-36,39,41-45)^ and 10 studies used DLQI^(16-20,26,28,31,33,46)^), followed by studies with head and neck cancer patients [5 studies used Skindex-16^(23-24,30,38,43)^ and 2 used DLQI^(32,46)^]. PSRQ^(21)^ was used in a study with breast cancer patients, as was Skindex-29^(37)^.

## Discussion

In this scoping review, four instruments used in cancer patients to assess skin-related quality of life were identified, namely: DLQI, Skindex-16, Skindex-29, and PRSQ. This is the first scoping review to map QoL instruments used to assess the skin in cancer patients who underwent radiotherapy and developed radiodermatitis.

Dermatological reactions generally affect individuals’ self-image and can lead to low self-esteem, social isolation, anxiety, and depression^(54-55)^. In addition, there are physical repercussions, such as pain, itching, and lesions, which make it impossible to perform daily activities^(56)^. This context affects individuals’ QoL, as it causes changes in functional, emotional, cultural, and psychosocial aspects^(56-58)^ and has a major influence on people’s well-being. Furthermore, QoL assessment facilitates the evaluation of therapeutic results from the patient’s perspective, the monitoring of the evolution of signs and symptoms, and the development of approaches for care focused on reducing negative impacts on the patient^(40,55,57-58)^.

With regard to radiodermatitis, the measurement of skin-specific QoL is fundamental in the evaluation process regarding how much this radiotoxicity affects the individual’s QoL. In a Brazilian study of 60 women, a 100% incidence of skin irritation from radiodermatitis was observed, and through the DLQI, it was possible to observe its impact mainly in the leisure domain^(54)^.

It was observed that, as treatment progressed and radiodermatitis appeared or worsened, there were negative repercussions on the well-being of these women and, consequently, on their QoL. Thus, the lack of data on the impacts of radiodermatitis on patients’ QoL prevents us from assessing the extent to which the condition can harm, for example, self-esteem and self-image. By evaluating such data, it is possible to develop comprehensive and individualized care according to each patient’s needs^(54)^.

Breast cancer patients were the most evaluated in terms of the impact of radiodermatitis on QoL^(15-28,30-31,33-37,39,41-46)^. It is considered essential that these instruments be evaluated for application in all cancer patients undergoing radiotherapy, assuming that they will develop some degree of radiodermatitis during treatment, regardless of the site being irradiated. This allows for the observation of the effects of this adverse reaction on patients’ quality of life and, thus, the planning of efficient care.

There is no consensus on which instrument to use to assess how much skin toxicity from radiotherapy impacts QoL^(54)^, but it is essential that scales/instruments/questionnaires be able to assess physical, emotional, and functional aspects. Skindex-16 assesses mental and emotional aspects more efficiently than DLQI. On the other hand, DLQI can assess issues such as daily functioning, clothing, sports, and difficulties encountered in treatment, which cannot be assessed by Skindex-16^(57)^.

Studies affirm^(25,57)^ that Skindex-16 provides more details regarding skin symptoms than other instruments such as DLQI. Skindex-16 is more recommended for individuals with minor changes in QoL, since DLQI is insensitive to minor impairments in QoL^(54)^. In addition, patient perception can be considered an important factor for controlling signs and symptoms over time, as it provides data that may not be observed by healthcare professionals^(41)^. Therefore, Skindex-16 has been used more to assess skin-related QoL in patients with radiodermatitis.

Another important point to consider when applying an instrument is its validation, since it adapts the questionnaire to the target audience—in this case, individuals in the Brazilian population with radiodermatitis. These tools are mostly developed according to the context of the country in which they are developed, considering its social, economic, and cultural context. Thus, when used in other countries, it is important that there be linguistic, cultural, and social adaptation that considers the new assessment scenario. Validation is also a more viable alternative than developing a new instrument, since it generates fewer costs and standardizes the information passed on by the scientific community, facilitating understanding and standardization of results^(59)^.

Therefore, in order to have a skin-related QoL instrument, it is essential to consider the appropriateness of the language for the target audience. It is also important to verify the reliability and comprehensiveness of the instrument, taking into account not only physical aspects, but also psychosocial and cultural aspects^(59)^, as an individual’s perception of a given factor may vary according to their country of origin, culture, beliefs, among other factors^(53)^. These tools provide patients’ perspectives on how dermatological conditions affect their daily lives.

Finally, the relevance of using instruments that measure patients’ dermatological QoL in the clinical practice of the nursing team should be considered. They are essential for promoting patient safety, as they reflect the patient’s own perspective on their health-disease process and, consequently, enable efficient communication not only among the team but also with the patient, in order to identify and prioritize issues that need to be addressed but have not been identified by professionals.

This scenario allows patients to be agents of their own care and to build strategies together with nurses to improve aspects of their QoL affected, for example, by radiodermatitis. The recording of these instruments can also assist nurses not only in the construction of institutional protocols, since common patterns can be observed among different patients, but also enables health promotion—effective health actions—and possible prophylaxis for certain problems that affect QoL.

This review provides greater knowledge about the instruments and questionnaires that assess skin-related QoL. Based on this, it contributes to decision-making about which instruments are most appropriate for assessing skin-related QoL in future studies. In addition to guiding clinical practice and the establishment of protocols in health institutions. Considering the impacts that radiodermatitis can have on the QoL of patients undergoing radiotherapy, this knowledge is fundamental for the provision of qualified care, covering all aspects of patient-centered care.

Among the limitations of this review, we can mention the limited number of scientific publications on the topic, which resulted in a small sample for data synthesis. Another limitation was that most studies presented QoL measurement as a secondary outcome of the studies, not presenting more robust data on the topic. In addition, these limitations make it difficult to reach a consensus on the most appropriate QOL measurement tool for use in the clinical practice of nurses who care for patients with radiodermatitis. This hinders a more detailed analysis of the nurse’s clinical findings and, consequently, prevents the development of more efficient protocols and care.

## Conclusion

Different instruments were identified that are used to assess skin-related QoL in patients with radiodermatitis, notably the Skindex-16 and the DLQI. The diversity of tools points to the absence of consolidated guidelines on the choice of the best instrument to be implemented in this clinical context. Thus, further studies are needed to evaluate the use of instruments to assess QoL in patients with radiodermatitis, aiming to assess which instrument is most suitable for this purpose, the frequency of application of the instruments, and how health professionals can act to improve the indices and promote an improvement in patients’ QoL.

## Data Availability

The dataset of this article is available on the RLAE page in the SciELO Data repository, at the link https://doi.org/10.48331/SCIELODATA.UBXFJ0.
