# Supplementary material for: Quality of life assessment instruments for radiodermatitis in cancer patients: scoping review
Source: Rev Lat Am Enfermagem. 2025 Nov 3;33:e4698. doi: 10.1590/1518-8345.7739.4698 (PMC12587760; doi:10.1590/1518-8345.7739.4698)
Supplement: Translation

# Translation: Instrumentos de avaliação de qualidade de vida para radiodermatite em pacientes oncológicos: revisão de escopo

**DOI:** 10.1590/1518-8345.7739.4699

**Authors:** Letícia Moraes Lopes, Beatriz Nappo Neiva, Beatriz Regina Lima de Aguiar, Paloma Gomes, Elaine Barros Ferreira, Paula Elaine Diniz dos Reis

**Affiliations:** 1 Universidade de Brasília, Faculdade de Ciências da Saúde, Brasília, DF, Brasil; 2 Bolsista da Fundação de Apoio à Pesquisa do Distrito Federal (FAPDF), Brasil; 3 Bolsista da Coordenação de Aperfeiçoamento de Pessoal de Nível Superior (CAPES), Brasil; 4 Bolsista do Conselho Nacional de Desenvolvimento Científico e Tecnológico (CNPq), Brasil

**Keywords:** Qualidade de Vida, Pele, Radiodermatite, Radioterapia, Neoplasias, Revisão

## Abstract

mapear na literatura os questionários e instrumentos de avaliação da Qualidade de Vida relacionados à pele, aplicados a pacientes com câncer submetidos à radioterapia que desenvolveram radiodermatite.

revisão de escopo conduzida de acordo com as recomendações do JBI. A busca foi realizada em sete fontes de informação e na literatura cinzenta. Não houve restrição quanto ao período de publicação nem ao idioma dos artigos. Foi realizada uma síntese qualitativa dos dados, apresentada de forma descritiva e por meio de tabelas e figuras.

trinta e dois estudos foram incluídos, sendo a maioria proveniente da América do Norte (n=14). Os instrumentos utilizados para avaliação da qualidade de vida relacionada à pele foram: o Índice de Qualidade de Vida em Dermatologia (n=17), o Skindex-16 (n=11), o Skindex-29 (n=1) e o *Padua Skin-Related Quality of Life* (n=1). Os instrumentos mais frequentemente aplicados foram o Índice de Qualidade de Vida em Dermatologia e o Skindex-16, utilizados predominantemente em pacientes com câncer de mama.

foram identificados diferentes instrumentos utilizados para avaliar a qualidade de vida relacionada à pele em pacientes com radiodermatite, com destaque para o Skindex-16 e o DLQI. A diversidade de ferramentas aponta para a ausência de diretrizes consolidadas sobre a escolha do melhor instrumento a ser implementado nesse contexto clínico.

## Destaques

**(1)** DLQI, Skindex-16, Skindex-19 e PRSQ são instrumentos específicos para radiodermatite. **(2)** Skindex-16 avalia aspectos mentais e emocionais de modo mais eficiente do que o DLQI. **(3)** A escolha do instrumento deve considerar as especificidades e necessidades da pele.

## Introdução

A radiodermatite é um evento adverso inflamatório que se manifesta com lesões cutâneas decorrentes da exposição à radiação ionizante^(1)^, com alta incidência nas regiões da pelve, mama, e cabeça e pescoço^(1)^. Em um estudo realizado no interior de São Paulo, Brasil, com mulheres que irradiaram a mama houve incidência de 98,2% de radiodermatite^(2)^. Na fase aguda, a radiodermatite é caracterizada pela presença de uma ou mais manifestações, como hiperpigmentação, eritema, descamação seca, descamação úmida, necrose, ulceração e infecção local^(1)^. Frequentemente, essa radiotoxicidade aparece a partir da segunda semana de tratamento^(3)^.

A gravidade da lesão tecidual que ocorre na radiodermatite pode variar de acordo com a dose por fração, a dose total administrada e a susceptibilidade individual, como idade, índice de massa corporal, exposição ao sol, fatores genéticos, dentre outros^(2,4-6)^. Além disso, quanto maior a gravidade da radiodermatite, maior a possibilidade de desconforto, impacto psicossocial, prejuízo nas atividades de vida diárias e interrupção do tratamento, o que pode afetar o prognóstico do paciente^(7)^. Todos esses fatores afetam a qualidade de vida dos pacientes^(8)^.

Assim, a qualidade de vida relacionada à saúde se refere à percepção que o indivíduo tem de sua própria condição de vida diante da doença, bem como do tratamento e evolução da enfermidade^(9)^. A sua avaliação em pacientes que apresentam algum grau de radiodermatite por meio do uso de instrumentos para sua mensuração permite a individualização dos cuidados e a consideração de fatores subjetivos, como o estado psicológico, as interações sociais e a autoestima^(10)^. Além disso, facilita o planejamento do cuidado, o acompanhamento de sinais, sintomas e efeitos colaterais, de modo que os profissionais possam promover o cuidado baseado nas queixas do paciente^(10)^.

A partir desse contexto, observa-se, cada vez mais, o uso de questionários e de escalas para avaliação dos diferentes contextos na área da saúde. Portanto, é imprescindível garantir a acurácia e a eficiência desses instrumentos^(11)^ para proporcionar a avaliação adequada dos resultados e promover um plano de ação eficaz. Além disso, é essencial identificar questionários e instrumentos que avaliem Qualidade de Vida (QV) que possam ser aplicados para a população brasileira, especificamente aqueles de origem nacional ou traduzidos para o português brasileiro.

A tradução dos instrumentos para o português brasileiro proporciona adaptação ao contexto cultural e linguístico do país e aumenta sua credibilidade^(10-11)^. Ademais, questionários específicos que avaliem a QV em pacientes com radiodermatite podem trazer maior confiabilidade e validade aos resultados. Esses fatores ressaltam a importância de identificar e analisar instrumentos voltados a essa finalidade, uma lacuna ainda não explorada na literatura. Uma busca preliminar abrangente realizada em plataformas de registro de protocolos de revisão, como o *Open Science Framework* (OSF) e o PROSPERO, além da base de dados *PubMed*, não identificou estudos publicados ou registrados com o mesmo tema.

Assim, o objetivo deste estudo foi mapear na literatura os questionários e os instrumentos de avaliação de QV relacionados à pele, aplicados a pacientes com câncer submetidos à radioterapia que desenvolveram radiodermatite.

## Método

### Protocolo e registro

Trata-se de uma revisão de escopo conduzida de acordo com as recomendações do JBI^(12)^. Para relatar esta revisão, utilizou-se o *checklistPreferred Reporting Items for Systematic Reviews and Meta Analyses* (PRISMA) *for Scoping Reviews* (PRISMA-ScR)^(13)^. O protocolo desta revisão de escopo foi registrado na plataforma *Open Science Framework* (OSF)^(14)^, sendo identificado pelo *Digital Object Identifier* (DOI) 10.17605/OSF.IO/UH42P e disponível publicamente por meio do endereço eletrônico: https://osf.io/uh42p/.

Esta revisão objetivou responder à pergunta norteadora: “Quais as evidências disponíveis na literatura acerca dos instrumentos utilizados para mensurar a QV relacionada à pele de pacientes adultos com câncer submetidos à radioterapia que desenvolveram radiodermatite?”. A elaboração desta pergunta foi feita por meio da utilização do acrônimo PCC, sendo P (população) – pacientes adultos com câncer submetidos à radioterapia; C (conceito) – questionários e instrumentos utilizados para mensurar a QV relacionada à pele de pacientes adultos com câncer submetidos à radioterapia que desenvolveram radiodermatite; C (contexto) – qualquer ambiente, seja ele hospitalar, ambulatorial ou domiciliar, nacional ou internacional, em que a QV relacionada à pele tenha sido avaliada durante a realização da radioterapia ou até quatro semanas após sua finalização.

### Critério de elegibilidade

Foram incluídos estudos observacionais e experimentais que mensuravam a QV relacionada à pele durante período transcorrido da radioterapia, com instrumentos validados ou não, em pacientes adultos com câncer submetidos à radioterapia que desenvolveram radiodermatite. Não houve restrição quanto ao período de publicação e nem quanto ao idioma dos artigos. Dessa forma, foram incluídos estudos de diferentes nacionalidades, abrangendo tanto publicações nacionais quanto internacionais, realizados em diversos contextos.

Foram excluídos os estudos que: (1) incluíram amostra composta por crianças e adolescentes (<18 anos); (2) estudos que não apresentaram avaliação de QV por meio de instrumentos; (3) estudos que utilizaram instrumentos para avaliação de QV não específicos para a pele; (4) estudos com pacientes que não realizaram radioterapia.

### Fontes de informação

Para identificação de estudos e descritores potencialmente relevantes, fez-se uma busca inicial na fonte de informação *PubMed*. Em seguida, uma estratégia de busca eletrônica foi projetada especificamente para o *PubMed* via *National Institutes of Health* (NIH) e posteriormente adaptada para cada uma das seguintes fontes de informação: *Latin American and Caribbean Literature on Health Sciences* (LILACS) via Biblioteca Virtual da Saúde (BVS), *Web of Science Core Collection* (WoSCC) via *Clarivate*; *Embase* via *Elsevier*, *LIVIVO* e *Cochrane Database* via *Cochrane Library*. Além disso, foram empreendidas pesquisas na literatura cinzenta por meio do *Google Scholar* e no *ProQuest* ^TM^ *Dissertation & Theses Citation Index* (*ProQuest*) via *Clarivate*.

Termos relacionados à população do acrônimo PCC não foram utilizados para não restringir a busca. A primeira estratégia de busca foi construída utilizando *MeSH Terms* e palavras-chave para uso na base de dados *PubMed.* Em seguida, as demais fontes de descritores – Descritores em Ciências da Saúde (DeCS) e *Emtree* – foram consultadas para adaptação da estratégia de busca para cada base de dados. Operadores booleanos (OR e AND) foram utilizados para combinar os termos. A busca que compôs os resultados desta revisão foi realizada em 25 de janeiro de 2024 em todas as fontes de informação e literatura cinzenta. As etapas de condução da revisão se deram entre janeiro e outubro de 2024.

Por fim, as listas de referências dos estudos incluídos também foram examinadas com o objetivo de identificar possíveis estudos relevantes que não tenham sido capturados na estratégia de busca inicial.

### Estratégia de busca

A estratégia de busca eletrônica completa utilizada no *PubMed*, acessada por meio da plataforma *National Institutes of Health* (NIH), foi a seguinte:

(“radiodermatitis”[MeSH Terms] OR “radiodermatitis”[All Fields] OR “radiation dermatitis”[All Fields] OR “radioepidermitis”[All Fields] OR “radiation reaction”[All Fields] OR “radio-epithelitis”[All Fields] OR “acute radiation reactions”[All Fields] OR “radiation-induced acute skin”[All Fields] OR “radio-induced damage”[All Fields] OR “cutaneous radiation syndrome”[All Fields] OR “radiodermatitides”[All Fields] OR “radiation-induced dermatitis”[All Fields] OR “radiation-induced skin lesions”[All Fields] OR “radiation induced acute toxicity”[All Fields] OR “radiation-induced toxicity”[All Fields] OR “radiation-induced toxicities”[All Fields] OR “radiation-induced normal tissue toxicity”[All Fields] OR “skin reaction”[All Fields] OR “skin reactions”[All Fields] OR “skin toxicity”[All Fields] OR “skin toxicities”[All Fields] OR “radiation-induced side effects”[All Fields] OR “radiation toxicity”[All Fields] OR “tissue complications”[All Fields] OR “radiation injury”[All Fields]) AND (“quality of life”[MeSH Terms] OR “quality of life”[All Fields] OR “health-related quality of life”[All Fields] OR “HRQL”[All Fields] OR “HOL”[All Fields] OR “hrqols”[All Fields] OR “quality of life”[All Fields] OR “hrqol”[All Fields] OR “health-related quality of life”[All Fields] OR “life quality”[All Fields]) AND (“questionnair”[All Fields] OR “questionnaire s”[All Fields] OR “surveys and questionnaires”[MeSH Terms] OR “surveys and questionnaires”[All Fields] OR “questionnaire”[All Fields] OR “questionnaires”[All Fields] OR “measurability”[All Fields] OR “measurable”[All Fields] OR “measurably”[All Fields] OR “measure s”[All Fields] OR “measureable”[All Fields] OR “measured”[All Fields] OR “measurement”[All Fields] OR “measurement s”[All Fields] OR “measurements”[All Fields] OR “measurer”[All Fields] OR “measurers”[All Fields] OR “measuring”[All Fields] OR “measurings”[All Fields] OR “measurement”[All Fields] OR “measurements”[All Fields] OR “weights and measures”[MeSH Terms] OR “instrument”[All Fields] OR “instrument s”[All Fields] OR “instrumentation”[All Fields] OR “instruments”[All Fields] OR “instrumented”[All Fields] OR “instrumenting”[All Fields] OR “surveys”[All Fields]).

### Seleção de fontes de evidência

Após a realização da identificação dos estudos nas fontes de informação, eles foram exportados para o gerenciador de referências *software EndNoteBasic* ^®^ (Thomson Reuters, EUA), versão *online*, para exclusão automática de duplicatas. Em seguida, as referências foram enviadas para o *softwareRayyan*, no qual se fez a avaliação dos títulos e dos resumos, de forma independente, por dois revisores (L.M.L. e B.N.N.). No processo, foram excluídos os estudos que não se enquadraram nos critérios de elegibilidade. Em seguida, as referências selecionadas foram lidas na íntegra pelos mesmos revisores e, novamente, aquelas que não atendiam aos critérios de elegibilidade foram excluídas desta revisão. Nas duas etapas, caso houvesse divergência entre os dois revisores sobre a inclusão de algum estudo, um terceiro revisor (P.E.D.R.), com expertise na temática, foi acionado para decidir sobre a inclusão ou exclusão do estudo na revisão. Os estudos selecionados seguiram para a fase de extração de dados. Foram selecionados artigos publicados entre 2004 e 2023.

### Processo de extração de dados e dados coletados

Para a extração de dados, utilizou-se uma tabela padronizada no *software Microsoft Excel*, elaborada pelos autores, contemplando informações relacionadas às publicações e aos domínios do PCC. A extração dos dados dos estudos incluídos foi realizada pelo primeiro revisor (L.M.L.) e conferida pelo segundo revisor (B.N.N.). Os seguintes dados foram coletados: características dos estudos (ano de publicação e país), características da amostra (tipo de câncer e presença de radiodermatite) e características dos instrumentos de avaliação de QV relacionada à saúde aplicados a pacientes com radiodermatite. Caso algum desses dados não estivesse disponível no estudo, procedia-se ao envio de três *e-mails*, com intervalos de 15 dias, solicitando as referidas informações para o autor correspondente.

Além disso, inseriu-se nesta revisão uma avaliação detalhada dos instrumentos, baseada na coleta de dados da referência original do instrumento, para apresentar as seguintes informações: validação para língua portuguesa, itens que compõem o instrumento, escala e pontuação de cada item, forma de aplicação do instrumento, forma de avaliação do resultado obtido com o instrumento e tempo médio de resposta.

### Síntese dos resultados

O desfecho primário desta revisão de escopo foi a descrição dos instrumentos utilizados para a avaliação de QV relacionada à pele em pacientes com câncer submetidos à radioterapia que apresentaram radiodermatite. Adicionalmente, verificou-se o número de estudos publicados por ano, a distribuição do uso dos instrumentos de avaliação de QV por região geográfica, a característica dos instrumentos que mensuraram QV e a frequência dos instrumentos usados por tipo de câncer. Os dados foram analisados por meio de síntese qualitativa e apresentados de forma descritiva e por meio de tabelas e figuras.

### Aspectos éticos

Por se tratar de um estudo secundário que utiliza como fonte de dados estudo primários já publicados, este trabalho não necessita de apreciação pelo Comitê de Ética em Pesquisa.

## Resultados

### Seleção dos estudos

Foram identificadas 1.371 referências nas fontes de informação e, após remoção automática de estudos duplicados pelo *software Endnote*, restaram 1.198 estudos. Após a etapa mencionada, as referências foram encaminhadas para o *software Rayyan*, onde fez-se a remoção manual de mais 207 estudos duplicados. Seguiram para leitura de títulos e resumos: 991 referências. Após aplicar os critérios de elegibilidade, foram excluídos 919 estudos e 72 seguiram para leitura na íntegra. Vinte e três estudos de acesso restrito não foram recuperados para leitura na íntegra, mesmo após tentativa de contato com os autores. Os 49 estudos restantes, foram analisados quanto à sua elegibilidade, resultando em 24 estudos excluídos e 25 incluídos nesta revisão de escopo, advindos da busca nas fontes de informação.

A partir da literatura cinzenta e da busca manual na lista de referências dos estudos incluídos obtidos nas fontes de informação, identificamos 29 produções científicas, dentre as quais foram excluídas 7 duplicatas e 13 não foram recuperadas. Para os estudos não disponíveis na íntegra, foram enviados três e-mails, com intervalos de 15 dias, solicitando as informações para o autor correspondente. Em todas as tentativas, não se obteve resposta. Assim, 9 estudos seguiram para a etapa de avaliação de títulos, resumos e, depois, para a leitura na íntegra. Após aplicar os critérios de elegibilidade, 7 estudos foram incluídos na revisão advindos da busca por outras fontes.

Desse modo, um total de 32 estudos foram incluídos nesta revisão de escopo^(15-46)^. Os processos de busca e seleção dos estudos foram detalhados na Figura 1.

**Figura 1-:** Fluxograma do processo de identificação, triagem e inclusão de estudos. Adaptado de PRISMA (2020)^(47)^

### Características dos estudos

Todos os estudos incluídos foram publicados em inglês, entre os anos de 2004 e 2023, com um pico de publicações em 2018 (5 estudos), seguido pelos anos de 2019 e 2022, com 4 estudos cada (Figura 2).

**Figura 2-:** Número de estudos incluídos por ano de publicação

No que se refere ao continente de origem das publicações, a América do Norte apresenta o maior número de estudos (n = 14; 43,75%), todos realizados nos Estados Unidos da América (EUA)^(16-21,27,29,34,36,41-44)^. Em seguida, a Europa, com 7 publicações, sendo 4 da Bélgica^(22,37-39)^, 2 do Reino Unido^(28,46)^ e 1 da Itália^(30)^. A Ásia reúne 6 estudos, dos quais 4 são do Irã^(31-33,35)^, 1 do Japão^(45)^ e 1 da Tailândia^(25)^. A Oceania conta com 2 estudos, ambos da Austrália^(23-24)^. A América do Sul também apresenta 2 estudos, ambos provenientes do Brasil^(26,40)^. Por fim, a África apresenta 1 estudo, originário do Egito^(15)^.

### Resultados dos estudos individuais

Os instrumentos de QV relacionada à pele identificados foram: o Índice de Qualidade de Vida em Dermatologia (do inglês *Dermatology Life Quality Index* (DLQI))^(16-20,26,28,31-33,46)^, o Skindex-16^(15,22-25,27,29-30,34-36,38-39,41-45)^, o Skindex-29^(37)^ e o *Padua Skin-Related Quality of Life* (PSRQ)^(21)^. O Skindex-16 foi relatado em estudos desenvolvidos nos EUA (n=8; 25%), Bélgica (n=3; 9,375%), Austrália (n=2; 6,25%), Itália (n=1; 3,125%), Tailândia (n=1; 3,125%), Irã (n=1; 3,125%) e Egito (n=1; 3,125%). Já o DLQI foi relatado em estudos desenvolvidos nos EUA (n=5; 15,625%), Irã (n=3; 9,375%), Reino Unido (n=2; 6,25%) e Brasil (n=1; 3,125%). Os instrumentos Skindex-29 e o PSRQ foram relatados em 1 estudo cada, sendo conduzidos na Bélgica e nos EUA, respectivamente.

Dessa forma, observa-se predomínio do uso do Skindex-16 nos continentes da África, América do Norte, Europa e Oceania, enquanto o DLQI predomina na América do Sul e na Ásia (Figura 3). As revisões sistemáticas, uma do Brasil^(40)^ e outra do Japão^(45)^, não foram incluídas na Figura 3 para que não houvesse contabilização duplicada de estudos primários.

O instrumento DLQI^(48)^ tem por objetivo quantificar o quanto uma afecção de pele afeta a QV de uma pessoa. Trata-se de um questionário composto por 10 itens divididos em 6 categorias, sendo elas: sintomas e sentimentos (2 itens), atividade diária (2 itens), lazer (2 itens), trabalho e estudo (1 item), relações interpessoais (2 itens) e tratamento (1 item). Cada item permite uma atribuição de 0 (nada) a 3 (muitíssimo) pontos, podendo a somatória total variar entre 0 e 30 pontos. Assim como em outros questionários, é importante analisar o contexto sociocultural e econômico da população, visto que se pode observar diferentes ênfases nas perguntas respondidas.

**Figura 3-:** Frequência dos Instrumentos de Qualidade de Vida relacionada à pele em pacientes com radiodermatite por continente

O Skindex^(49)^ consiste em um instrumento de avaliação da QV dermatológica, cujo objetivo é mensurar o impacto de afecções dermatológicas e seus efeitos na QV dos indivíduos. Ademais, possibilita distinguir as diferentes manifestações cutâneas – como psoríase, melasma, úlceras crônicas, vitiligo, rosáceas, dentre outros – e acompanhar sua evolução ao longo de um período. Nele, são avaliados 3 domínios, a saber: funcional, emoções e sintomas.

Nesta revisão, foram identificados o Skindex-16^(15,22-25,27,29-30,34-36,38-39,41-45)^ e o Skindex-29^(37)^, os quais possuem especificidades em comum. Em ambos os questionários, o item é desconsiderado quando para ele houver mais de uma resposta. Para estes instrumentos, é possível quantificar cada domínio ao fazer a pontuação média das respostas, por meio de uma pontuação de três escalas. Ou seja, cada domínio possui uma média e são avaliados separadamente um do outro.

O Skindex-29^(49)^ é composto por 29 itens divididos entre os domínios das emoções (10 itens), dos sintomas (7 itens) e funcional (12 itens). A atribuição da pontuação é de 1 ponto para “nunca” e 5 pontos para “o tempo todo”. A pontuação do Skindex-29 é então transferida para uma escala linear, a qual varia de 0 a 100 para cada domínio, em que quanto mais alta é a pontuação, maior é o efeito sobre a QV do indivíduo. O escore total para a avaliação da QV pode resultar em 0 pontos (nunca), 25 pontos (raramente), 50 pontos (às vezes), 75 pontos (frequentemente) e 100 pontos (o tempo todo). A pontuação final consiste na pontuação média de cada domínio, o corte para identificar pacientes com QV gravemente prejudicada é ≥ 52 para o domínio dos sintomas, ≥ 39 para o domínio das emoções, ≥ 37 para o domínio funcional e ≥ 44 para o escore total.

O Skindex-16^(49)^ possui 16 itens, dentre os quais 7 são para o domínio de emoções, 4 para sintomas e 5 para o funcional. O instrumento foi desenvolvido a partir do Skindex-29 com o intuito de ser mais sucinto, preciso e eficiente. Sua construção se baseou na manutenção de itens que apresentaram melhores desempenhos no Skindex-29 e na adição de novas perguntas. A escala utilizada é do tipo Likert e a pontuação de seus itens varia de 0 pontos para “nunca incomodou” a 6 pontos para “sempre incomodou”. O escore total de cada item também é transformado em uma escala linear de 0 a 100 pontos e a pontuação de cada domínio do Skindex-16 consiste na média de seus itens.

Por último, o PSRQ^(50)^ é um questionário italiano, concebido inicialmente com 63 itens, com intuito de mensurar tanto o nível de conforto quanto de desconforto relacionado à pele. Atualmente, o PSRQ é composto por 50 itens, sendo que cada item permite uma resposta que varia entre 1 “discordo totalmente” a 5 “concordo plenamente”. O instrumento apresenta 5 domínios, sendo eles comprometimento interpessoal (12 itens), sentimentos e emoções positivas (17 itens), sentimentos e emoções negativas (13 itens), estresse e comprometimento físico (8 itens). Seu resultado pode ser obtido pela somatória total de todos os itens ou por domínio, por meio da somatória da pontuação média obtida pelas respostas de cada conjunto de itens que formam o domínio.

Para todos os instrumentos apresentados, quanto maior a pontuação, maior o comprometimento da QV relacionada à pele dos indivíduos. As principais características dos instrumentos são apresentadas na Figura 4.

**Figura 4-:** Características dos Instrumentos de Qualidade de Vida relacionada à pele identificados nos artigos identificados nos artigos selecionados (n = 32). Brasília, DF, Brasil, 2024

Instrumento	Validado para língua portuguesa	Número de itens	Número de categorias	Nome das categorias	Pontuação	Pontuação atribuída ao item	Tempo de resposta em minutos
Índice de Qualidade de Vida em Dermatologia (DLQI) ^( 48 , 51 )^	Sim	10	6	1.Sintomas e sentimentos	Máxima: 30 / Mínima:0	0 a 3 pontos por cada item	Aproximadamente 2
				2.Atividade diária		0.Nada	
				3.Lazer		1.Um pouco	
				4.Trabalho e estudo		2.Muito	
				5.Relações interpessoais		3.Muitíssimo	
				6.Tratamento			
Skindex-16 ^( 49 , 52 )^	Sim	16	3	1.Emoções	Máxima: 96 / Mínima: 0	0 a 6 pontos por cada item	2-3
				2.Atividades diárias		0.Nunca incomodou	
				3.Sintomas		6.Sempre incomodou	
Skindex-29 ^( 49 , 53 )^	Sim	29	3	1.Emoções	Máxima: o tempo todo/ Mínima: nunca	1.Nunca	Aproximadamente 5
				2.Atividades diárias		2.Raramente	
				3.Sintomas		3.Às vezes	
						4.Frequentemente	
						5.O tempo todo	
*Padua Skin-Related QoL Questionnaire* ^( 50 )^	Não	50	5	1.Comprometimento interpessoal	Máxima: 250/ Mínima: 50	1 a 5 pontos por cada item	-
				2.Sentimentos e emoções positivas		1.Discordo totalmente	
				3.Sentimentos e emoções negativas		5. Concordo totalmente	
				4.Estresse			
				5.Comprometimento físico			

O Skindex-16 e o DLQI foram os instrumentos mais utilizados para avaliar a QV relacionada à radiodermatite (Figura 5). No que concerne ao tipo de câncer dos participantes dos estudos incluídos, verifica-se que a QV foi avaliada majoritariamente em indivíduos com câncer de mama (16 estudos utilizaram o Skindex-16^(15,22-25,27,30,34-36,39,41-45)^ e 10 estudos utilizaram o DLQI^(16-20,26,28,31,33,46)^), seguidos pelos estudos em pacientes com câncer de cabeça e pescoço [5 estudos utilizaram o Skindex-16^(23-24,30,38,43)^ e 2 utilizaram o DLQI^(32,46)^]. O PSRQ^(21)^ foi utilizado em um estudo com pacientes com câncer de mama, assim como o Skindex-29^(37)^.

**Figura 5–:** Frequência dos tipos de câncer apresentados pelos pacientes nos estudos que utilizaram o Skindex-16 e o *Dermatology Life Quality Index* (DLQI)

## Discussão

Nesta revisão de escopo, foram identificados quatro instrumentos utilizados em pacientes com câncer para avaliar a qualidade de vida relacionada à pele, a saber: DLQI, Skindex-16, Skindex-29 e PRSQ. Trata-se da primeira revisão de escopo que mapeia instrumentos de QV utilizados para avaliação da pele em pacientes com câncer que foram submetidos à radioterapia e desenvolveram radiodermatite.

Reações dermatológicas geralmente afetam a autoimagem dos indivíduos e podem ocasionar, baixa autoestima, isolamento social, ansiedade e depressão^(54-55)^. Além disso, há as repercussões físicas, como dor, prurido e lesões, que impossibilitam a realização de atividades do cotidiano^(56)^. Tal contexto afeta a QV dos indivíduos, visto que causa alterações nos aspectos funcional, emocional, cultural e psicossocial^(56-58)^ e possui grande influência no bem-estar das pessoas. Ademais, a avaliação da QV favorece a avaliação dos resultados terapêuticos sob a ótica do paciente, o monitoramento da evolução dos sinais e sintomas, e a elaboração de condutas para um atendimento centrado na diminuição dos impactos negativos sobre o paciente^(40,55,57-58)^.

No que tange à radiodermatite, a mensuração da QV específica para pele é fundamental no processo avaliativo referente ao quanto a radiotoxicidade afeta a QV do indivíduo. Em estudo brasileiro com 60 mulheres, observou-se a incidência de 100% de irritação da pele oriunda da radiodermatite e, por meio do DLQI, foi possível observar seu impacto principalmente no domínio lazer^(54)^.

Nele, foi observado que, à medida que o tratamento prosseguiu e a radiodermatite surgiu ou agravou, houve repercussões negativas no bem-estar das mulheres e, consequentemente, na QV. Assim, a falta de dados acerca dos impactos da radiodermatite na QV dos pacientes, impede dimensionar o quanto a condição pode prejudicar, por exemplo, a autoestima e a autoimagem. Ao avaliar tais dados, há possiblidade de desenvolver um cuidado integral e individualizado de acordo com a demanda de cada paciente^(54)^.

Pacientes com câncer de mama foram os mais avaliados quanto ao impacto da radiodermatite na QV^(15-28,30-31,33-37,39,41-46)^. Considera-se essencial que esses instrumentos sejam avaliados para aplicação em todos os pacientes com câncer submetidos à radioterapia, com pressuposto de que eles desenvolverão algum grau de radiodermatite durante o tratamento, independentemente do local que está sendo irradiado. Isso possibilita a observação dos efeitos dessa reação adversa na qualidade de vida dos pacientes e, assim, viabiliza o planejamento de um cuidado eficiente.

Não há consenso acerca de qual instrumento utilizar para avaliar o quanto a toxicidade da pele decorrente da radioterapia impacta a QV^(54)^, porém, é fundamental que escalas/instrumentos/questionários sejam capazes de avaliar aspectos físicos, emocionais e funcionais. O Skindex-16 avalia aspectos mentais e emocionais de modo mais eficiente do que o DLQI. Em contrapartida, o DLQI tem capacidade de avaliar questões como funcionamento diário, vestimenta, realização de esportes, dificuldades encontradas no tratamento, o que não pode ser avaliado pelo Skindex-16^(57)^.

Estudos afirmam^(25,57)^ que o Skindex-16 possui mais detalhes referentes aos sintomas cutâneos do que outros instrumentos como o DLQI. O Skindex-16 é mais recomendado para indivíduos com pequenas alterações na QV, visto que o DLQI é insensível para prejuízos pequenos na QV^(54)^. Além disso, a percepção dos pacientes pode ser considerado um fator importante para o controle de sinais e sintomas ao longo do tempo, uma vez que fornece dados que podem não ser observados pelo profissional de saúde^(41)^. Logo, o Skindex-16 tem sido mais utilizado para avaliar QV relacionada à pele em pacientes com radiodermatite.

Outro ponto importante que deve ser considerado ao se aplicar um instrumento é sua validação, visto que ela adequa o questionário ao público-alvo - no caso, indivíduos da população brasileira com radiodermatite. Essas ferramentas majoritariamente são elaboradas de acordo com o contexto do país em que é elaborado, considerando seu contexto social, econômico, cultural. Assim, ao serem utilizadas em outros países, é importante que haja uma adaptação linguística, cultural, social, que considere o novo cenário de avaliação. A validação é também uma alternativa mais viável do que a elaboração de um novo instrumento, uma vez que gera menos custos e padroniza as informações repassadas pela comunidade científica, facilitando a compreensão e a uniformização de resultados^(59)^.

Dessa maneira, para se ter um instrumento de QV relacionada à pele é fundamental considerar a adequação da linguagem ao público-alvo. Também é importante verificar a confiabilidade e a abrangência do instrumento, levando em conta não apenas aspectos físicos, mas também psicossociais e culturais^(59)^, pois a percepção de um indivíduo sobre determinado fator pode variar de acordo com o país de origem, a cultura, crenças, entre outros fatores^(53)^. Essas ferramentas fornecem a perspectiva dos pacientes sobre como condições clínicas dermatológicas afetam a vida cotidiana.

Por fim, deve-se considerar a relevância da utilização dos instrumentos que mensuram a QV dermatológica dos pacientes na prática clínica da equipe de enfermagem. Eles são essenciais para promover a segurança do paciente, visto que refletem a perspectiva do próprio paciente sobre seu processo de saúde-doença e, por conseguinte, permite a comunicação eficiente não só entre a equipe, mas também com o paciente, de modo a identificar e priorizar questões que precisam ser abordadas, mas não foram identificadas pelos profissionais.

Tal cenário faz com que o paciente seja agente de seu próprio cuidado e construa estratégias juntamente com os enfermeiros para melhorar os aspectos da sua QV afetadas, por exemplo, pela radiodermatite. O registro desses instrumentos pode também auxiliar o enfermeiro não só na construção de protocolos institucionais, visto que pode ser observado padrões em comum entre diversos pacientes, mas também possibilita a promoção de saúde – ações de saúde eficazes - e uma possível profilaxia para determinada problemática que afeta a QV.

A presente revisão permite um maior conhecimento sobre os instrumentos e questionários que avaliam a QV relacionada à pele. A partir disso, contribui para a tomada de decisão acerca de quais instrumentos são mais adequados para avaliação da QV relacionada à pele em futuros estudos. Além de nortear a prática clínica e o estabelecimento de protocolos em instituições de saúde. Ao considerar os impactos que a radiodermatite pode ocasionar na QV dos pacientes submetidos à radioterapia, esse conhecimento é fundamental para prestação de uma assistência qualificada, que abrange todos os aspectos de cuidado voltado ao paciente.

Dentre as limitações desta revisão, pode-se citar o número limitado de produções científicas sobre a temática desenvolvida, o que resultou em uma amostra reduzida para a síntese de dados. Outro limitador foi que grande parte dos estudos apresentaram a mensuração da QV como desfecho secundário dos estudos, não apresentando dados mais robustos sobre a temática. Além disso, tais limitações dificultam a formação de um consenso acerca de um instrumento para mensuração da QV mais adequado para a utilização na prática clínica de enfermeiros que prestam assistência a pacientes com radiodermatite. Isso dificulta uma análise mais detalhada dos achados clínicos do enfermeiro e, consequentemente, impede a construção de protocolos e cuidados mais eficientes.

## Conclusão

Foram identificados diferentes instrumentos utilizados para avaliar a QV relacionada à pele em pacientes com radiodermatite, com destaque para o Skindex-16 e o DLQI. A diversidade de ferramentas aponta para a ausência de diretrizes consolidadas sobre a escolha do melhor instrumento a ser implementado nesse contexto clínico. Assim, mais estudos são necessários para avaliar o uso de instrumentos para avaliar a QV em pacientes com radiodermatite, visando avaliar qual instrumento mais se adequa para este fim, a periodicidade de aplicação dos instrumentos e como os profissionais de saúde podem atuar para melhorar os índices e favorecer uma melhora da QV dos pacientes.

## Data Availability

O conjunto de dados deste artigo está disponível na página da RLAE no repositório SciELO Data, no link https://doi.org/10.48331/SCIELODATA.UBXFJ0.

# Translation: Instrumentos de evaluación de la calidad de vida para radiodermitis en pacientes oncológicos: revisión de alcance

**DOI:** 10.1590/1518-8345.7739.4697

**Authors:** Letícia Moraes Lopes, Beatriz Nappo Neiva, Beatriz Regina Lima de Aguiar, Paloma Gomes, Elaine Barros Ferreira, Paula Elaine Diniz dos Reis

**Affiliations:** 1 Universidade de Brasília, Faculdade de Ciências da Saúde, Brasília, DF, Brasil; 2 Becaria de la Fundação de Apoio à Pesquisa do Distrito Federal (FAPDF), Brasil; 3 Becaria de la Coordenação de Aperfeiçoamento de Pessoal de Nível Superior (CAPES), Brasil; 4 Becaria del Conselho Nacional de Desenvolvimento Científico e Tecnológico (CNPq), Brasil

**Keywords:** Calidad de Vida, Piel, Radiodermitis, Radioterapia, Neoplasias, Revisión

## Abstract

mapear en la literatura los cuestionarios e instrumentos de evaluación de la calidad de vida relacionada con la piel aplicados a pacientes oncológicos sometidos a radioterapia que desarrollaron radiodermitis.

se realizó una revisión de alcance de acuerdo con las recomendaciones del JBI, con búsquedas en siete fuentes de información y en literatura gris sin restricciones de periodo ni idioma, y se efectuó una síntesis cualitativa de los datos presentada de forma descriptiva mediante tablas y figuras.

se incluyeron 32 estudios, la mayoría procedentes de América del Norte (n = 14); los instrumentos utilizados fueron el Índice de Calidad de Vida en Dermatología (DLQI) (n = 17), el Skindex-16 (n = 11), el Skindex-29 (n = 1) y el *Padua Skin-Related Quality of Life* (PRSQ) (n = 1), siendo DLQI y Skindex-16 los más aplicados, predominantemente en pacientes con cáncer de mama.

existe una diversidad de instrumentos para evaluar la calidad de vida relacionada con la piel en pacientes con radiodermitis, destacándose Skindex-16 y DLQI, y la variedad de herramientas evidencia la ausencia de directrices consolidadas sobre la elección del instrumento óptimo para este contexto clínico.

## Destacados

**(1)** DLQI, Skindex-16, Skindex-19 y PRSQ son instrumentos específicos para radiodermitis. **(2)** Skindex-16 evalúa los aspectos mentales y emocionales con mayor eficacia que el DLQI. **(3)** La elección del instrumento debe tener en cuenta las especificidades y necesidades de la piel.

## Introducción

La radiodermatitis es un evento adverso inflamatorio que se manifiesta con lesiones cutáneas derivadas de la exposición a la radiación ionizante^(1)^, con alta incidencia en las regiones de la pelvis, mama, cabeza y cuello^(1)^. En un estudio realizado en el interior de São Paulo, Brasil, con mujeres que recibieron irradiación en la mama, se observó una incidencia del 98,2% de radiodermatitis^(2)^. En la fase aguda, la radiodermatitis se caracteriza por la presencia de una o más manifestaciones, como hiperpigmentación, eritema, descamación seca, descamación húmeda, necrosis, ulceración e infección local^(1)^. Frecuentemente, esta radiotoxicidad aparece a partir de la segunda semana de tratamiento^(3)^.

La gravedad de la lesión tisular que ocurre en la radiodermatitis puede variar de acuerdo con la dosis por fracción, la dosis total administrada y la susceptibilidad individual, como la edad, el índice de masa corporal, la exposición al sol, factores genéticos, entre otros^(2,4-6)^. Además, cuanto mayor es la gravedad de la radiodermatitis, mayor es la posibilidad de incomodidad, impacto psicosocial, perjuicio en las actividades de la vida diaria e interrupción del tratamiento, lo que puede afectar el pronóstico del paciente^(7)^. Todos estos factores afectan la calidad de vida de los pacientes^(8)^.

Así, la calidad de vida relacionada con la salud se refiere a la percepción que el individuo tiene de su propia condición de vida frente a la enfermedad, así como del tratamiento y la evolución de la afección^(9)^. Su evaluación en pacientes que presentan algún grado de radiodermatitis mediante el uso de instrumentos para su medición permite la individualización de los cuidados y la consideración de factores subjetivos, como el estado psicológico, las interacciones sociales y la autoestima^(10)^. Además, facilita la planificación del cuidado, el seguimiento de señales, síntomas y efectos colaterales, de modo que los profesionales puedan promover una atención basada en las quejas del paciente^(10)^.

En este contexto, se observa, cada vez más, el uso de cuestionarios y escalas para la evaluación de los diferentes contextos en el área de la salud. Por lo tanto, es imprescindible garantizar la precisión y la eficacia de estos instrumentos^(11)^ para proporcionar una evaluación adecuada de los resultados y promover un plan de acción eficaz. Además, es esencial identificar cuestionarios e instrumentos que evalúen la Calidad de Vida (CV) que puedan ser aplicados a la población brasileña, específicamente aquellos de origen nacional o traducidos al portugués brasileño.

La traducción de los instrumentos al portugués brasileño proporciona una adaptación al contexto cultural y lingüístico del país y aumenta su credibilidad^(10-11)^. Asimismo, cuestionarios específicos que evalúen la CV en pacientes con radiodermatitis pueden aportar mayor confiabilidad y validez a los resultados. Estos factores resaltan la importancia de identificar y analizar instrumentos orientados a este propósito, una laguna aún no explorada en la literatura. Una búsqueda preliminar exhaustiva realizada en plataformas de registro de protocolos de revisión, como *Open Science Framework* (OSF) y PROSPERO, además de la base de datos PubMed, no identificó estudios publicados o registrados con el mismo tema.

Así, el objetivo de este estudio fue mapear en la literatura los cuestionarios y los instrumentos de evaluación de la CV relacionados con la piel, aplicados a pacientes con cáncer sometidos a radioterapia que desarrollaron radiodermatitis.

## Método

### Protocolo y registro

Se trata de una revisión de alcance conducida de acuerdo con las recomendaciones del JBI^(12)^. Para el reporte de esta revisión se utilizó el *checklist Preferred Reporting Items for Systematic Reviews and Meta Analyses* (PRISMA) *for Scoping Reviews* (PRISMA-ScR)^(13)^. El protocolo de esta revisión de alcance fue registrado en la plataforma *Open Science Framework* (OSF)^(14)^ y está identificado por el *Digital Object Identifier* (DOI) 10.17605/OSF.IO/UH42P. El protocolo está disponible públicamente en la siguiente dirección electrónica: https://osf.io/uh42p/.

Esta revisión tuvo como objetivo responder a la pregunta orientadora: “¿Cuáles son las evidencias disponibles en la literatura acerca de los instrumentos utilizados para medir la CV relacionada con la piel de pacientes adultos con cáncer sometidos a radioterapia que desarrollaron radiodermatitis?”. La elaboración de esta pregunta se realizó mediante el uso del acrónimo PCC, siendo P (población) – pacientes adultos con cáncer sometidos a radioterapia, C (concepto) – cuestionarios e instrumentos utilizados para medir la CV relacionada con la piel de pacientes adultos con cáncer sometidos a radioterapia que desarrollaron radiodermatitis, y C (contexto) – cualquier entorno, ya sea hospitalario, ambulatorio o domiciliario, nacional o internacional, en el que se haya evaluado la CV relacionada con la piel durante la realización de la radioterapia o hasta 4 semanas después de su finalización.

### Criterio de elegibilidad

Se incluyeron estudios observacionales y experimentales que midieron la CV relacionada con la piel durante el período de realización de la radioterapia, con instrumentos validados o no, en pacientes adultos con cáncer sometidos a radioterapia que desarrollaron radiodermatitis. No hubo restricción con respecto al período de publicación ni al idioma de los artículos. De esta forma, se incluyeron estudios de diferentes nacionalidades, abarcando tanto publicaciones nacionales como internacionales, realizados en diversos contextos.

Se excluyeron los estudios que: (1) incluyeron muestra compuesta por niños y adolescentes (<18 años); (2) estudios que no presentaron evaluación de la CV mediante instrumentos; (3) estudios que utilizaron instrumentos para evaluación de la CV no específicos para la piel; (4) estudios con pacientes que no realizaron radioterapia.

### Fuentes de información

Para la identificación de estudios y descriptores potencialmente relevantes, se realizó inicialmente una búsqueda preliminar en la fuente de información *PubMed*. A continuación, se diseñó una estrategia de búsqueda electrónica específicamente para *PubMed* por intermedio del *National Institutes of Health* (NIH) y posteriormente se adaptó para cada una de las siguientes fuentes de información: *Latin American and Caribbean Literature on Health Sciences* (LILACS) vía *Biblioteca Virtual da Saúde* (BVS), *Web of Science Core Collection* (WoSCC) vía *Clarivate*; *Embase* vía *Elsevier*, LIVIVO y *Cochrane Database* vía *Cochrane Library*. Además, se realizaron búsquedas en literatura gris mediante el *Google Scholar* y en el *ProQuest* ^TM^ *Dissertation & Theses Citation Index* (ProQuest) vía *Clarivate*.

No se utilizaron términos relacionados con la población del acrónimo PCC para no restringir la búsqueda. La primera estrategia de búsqueda fue construida utilizando *MeSH Terms* y palabras clave para uso en la base de datos *PubMed*. Posteriormente, se consultaron otras fuentes de descriptores – Descriptores en Ciencias de la Salud (DeCS) y Emtree – para la adaptación de la estrategia de búsqueda a cada base de datos. Se utilizaron operadores booleanos (*OR* y *AND*) para combinar los términos. La búsqueda que compuso los resultados de esta revisión fue realizada el 25 de enero de 2024 en todas las fuentes de información y literatura gris. Las etapas de conducción de la revisión se llevaron a cabo entre enero y octubre de 2024.

Por último, también se examinaron las listas de referencias de los estudios incluidos con el objetivo de identificar posibles estudios relevantes que no hubieran sido capturados en la estrategia de búsqueda inicial.

### Estrategia de búsqueda

La estrategia de búsqueda electrónica completa utilizada en PubMed, accesada mediante la plataforma *National Institutes of Health* (NIH), fue la siguiente: (“radiodermatitis”[MeSH Terms] OR “radiodermatitis”[All Fields] OR “radiation dermatitis”[All Fields] OR “radioepidermitis”[All Fields] OR “radiation reaction”[All Fields] OR “radio-epithelitis”[All Fields] OR “acute radiation reactions”[All Fields] OR “radiation-induced acute skin”[All Fields] OR “radio-induced damage”[All Fields] OR “cutaneous radiation syndrome”[All Fields] OR “radiodermatitides”[All Fields] OR “radiation-induced dermatitis”[All Fields] OR “radiation-induced skin lesions”[All Fields] OR “radiation induced acute toxicity”[All Fields] OR “radiation-induced toxicity”[All Fields] OR “radiation-induced toxicities”[All Fields] OR “radiation-induced normal tissue toxicity”[All Fields] OR “skin reaction”[All Fields] OR “skin reactions”[All Fields] OR “skin toxicity”[All Fields] OR “skin toxicities”[All Fields] OR “radiation-induced side effects”[All Fields] OR “radiation toxicity”[All Fields] OR “tissue complications”[All Fields] OR “radiation injury”[All Fields]) AND (“quality of life”[MeSH Terms] OR “quality of life”[All Fields] OR “health-related quality of life”[All Fields] OR “HRQL”[All Fields] OR “HOL”[All Fields] OR “hrqols”[All Fields] OR “quality of life”[All Fields] OR “hrqol”[All Fields] OR “health-related quality of life”[All Fields] OR “life quality”[All Fields]) AND (“questionnair”[All Fields] OR “questionnaire s”[All Fields] OR “surveys and questionnaires”[MeSH Terms] OR “surveys and questionnaires”[All Fields] OR “questionnaire”[All Fields] OR “questionnaires”[All Fields] OR “measurability”[All Fields] OR “measurable”[All Fields] OR “measurably”[All Fields] OR “measure s”[All Fields] OR “measureable”[All Fields] OR “measured”[All Fields] OR “measurement”[All Fields] OR “measurement s”[All Fields] OR “measurements”[All Fields] OR “measurer”[All Fields] OR “measurers”[All Fields] OR “measuring”[All Fields] OR “measurings”[All Fields] OR “measurement”[All Fields] OR “measurements”[All Fields] OR “weights and measures”[MeSH Terms] OR “instrument”[All Fields] OR “instrument s”[All Fields] OR “instrumentation”[All Fields] OR “instruments”[All Fields] OR “instrumented”[All Fields] OR “instrumenting”[All Fields] OR “surveys”[All Fields]).

### Selección de fuentes de evidencia

Tras la identificación de los estudios en las fuentes de información, estos fueron exportados al gestor de referencias *EndNoteBasic* ^®^ (*Thomson Reuters*, EE. UU.), versión en línea, para la exclusión automática de duplicados. A continuación, las referencias fueron enviadas al *software Rayyan*, en el cual se realizó la evaluación de los títulos y resúmenes, de forma independiente, por dos revisores (L.M.L. y B.N.N.). En este proceso, se excluyeron los estudios que no cumplían con los criterios de elegibilidad. Luego, las referencias seleccionadas fueron leídas en su totalidad por los mismos revisores y, nuevamente, aquellas que no cumplían con los criterios de elegibilidad fueron excluidas de esta revisión. En ambas etapas, en caso de desacuerdo entre los dos revisores sobre la inclusión de algún estudio, se convocó a un tercer revisor (P.E.D.R.), con experiencia en la temática, para decidir sobre la inclusión o exclusión del estudio en esta revisión. Los estudios seleccionados pasaron a la fase de extracción de datos. Se seleccionaron artículos publicados entre 2004 y 2023.

### Proceso de extracción de datos y datos recolectados

Para la extracción de datos, se utilizó una tabla estandarizada en el *software Microsoft Excel*, elaborada por los autores, que contemplaba información relacionada con las publicaciones y los dominios del PCC. La extracción de los datos de los estudios incluidos fue realizada por el primer revisor (L.M.L.) y verificada por el segundo revisor (B.N.N.). Se recolectaron los siguientes datos: características de los estudios (año de publicación y país), características de la muestra (tipo de cáncer y presencia de radiodermatitis) y características de los instrumentos de evaluación de la CV relacionada con la salud aplicados a pacientes con radiodermatitis. En caso de que alguno de estos datos no estuviera disponible en el estudio, se enviaron tres correos electrónicos, con intervalos de 15 días, solicitando la información al autor correspondiente.

Además, se incluyó en esta revisión una evaluación detallada de los instrumentos, basada en la recopilación de datos de la referencia original del instrumento, para presentar la siguiente información: validación al idioma portugués, ítems que componen el instrumento, escala y puntuación de cada ítem, forma de aplicación del instrumento, forma de evaluación del resultado obtenido con el instrumento y tiempo medio de respuesta.

### Síntesis de los resultados

El desenlace primario de esta revisión de alcance fue la descripción de los instrumentos utilizados para la evaluación de la CV relacionada con la piel en pacientes con cáncer sometidos a radioterapia que presentaron radiodermatitis. Adicionalmente, se verificó el número de estudios publicados por año, la distribución del uso de los instrumentos de evaluación de la CV por región geográfica, la característica de los instrumentos que midieron la CV y la frecuencia de los instrumentos utilizados según el tipo de cáncer. Los datos fueron analizados mediante síntesis cualitativa y presentados de forma descriptiva y mediante tablas y figuras.

### Aspectos éticos

Por tratarse de un estudio secundario que utiliza como fuente de datos estudios primarios ya publicados, este trabajo no requiere aprobación por parte del Comité de Ética en Investigación.

## Resultados

### Selección de los estudios

Se identificaron 1.371 referencias en las fuentes de información y, tras la eliminación automática de estudios duplicados mediante el *software Endnote*, quedaron 1.198 estudios. Luego de esta etapa, las referencias fueron enviadas al *software Rayyan*, donde se eliminaron manualmente otros 207 estudios duplicados. Se procedió a la lectura de títulos y resúmenes de 991 referencias. Tras aplicar los criterios de elegibilidad, se excluyeron 919 estudios y 72 pasaron a lectura completa. Veintitrés estudios de acceso restringido no fueron recuperados para lectura completa, incluso después de intentos de contacto con los autores. Los 49 estudios restantes fueron analizados en cuanto a su elegibilidad, resultando en 24 estudios excluidos y 25 incluidos en esta revisión de alcance provenientes de la búsqueda en las fuentes de información.

A partir de la literatura gris y de la búsqueda manual en las listas de referencias de los estudios incluidos obtenidos en las fuentes de información, se identificaron 29 producciones científicas, de las cuales se excluyeron 7 duplicadas y 13 no fueron recuperadas. Para los estudios no disponibles en texto completo, se enviaron tres correos electrónicos, con intervalos de 15 días, solicitando la información al autor correspondiente. En todos los intentos, no se obtuvo respuesta. Así, 9 estudios pasaron a la etapa de evaluación de títulos y resúmenes y luego a lectura completa. Tras aplicar los criterios de elegibilidad, 7 estudios fueron incluidos en la revisión provenientes de la búsqueda en otras fuentes.

De este modo, un total de 32 estudios fueron incluidos en esta revisión de alcance^(15-46)^. El proceso de búsqueda y selección de los estudios fue detallado en la Figura 1.

**Figura 1-:** Diagrama de flujo del proceso de identificación, selección e inclusión de estudios. Adaptado de PRISMA (2020)^(47)^

### Características de los estudios

Todos los estudios incluidos fueron publicados en inglés, entre los años 2004 y 2023, con un pico de publicaciones en 2018 (5 estudios), seguido por los años 2019 y 2022, con 4 estudios cada uno (Figura 2).

**Figura 2-:** Numero de estudios incluidos en esta revisión de alcance por año de publicación

En cuanto al continente de origen de las publicaciones, América del Norte presenta el mayor número de estudios (n = 14; 43,75%), todos realizados en los Estados Unidos de América (EE. UU.)^(16-21,27,29,34,36,41-44)^. Le sigue Europa, con 7 publicaciones, siendo 4 de Bélgica^(22,37-39)^, 2 del Reino Unido^(28,46)^ y 1 de Italia^(30)^. Asia reúne 6 estudios, de los cuales 4 son de Irán^(31-33,35)^, 1 de Japón^(45)^ y 1 de Tailandia^(25)^. Oceanía cuenta con 2 estudios, ambos de Australia^(23-24)^. América del Sur también posee 2 estudios, ambos provenientes de Brasil^(26,40)^. Por último, África presenta 1 estudio, originario de Egipto^(15)^.

### Resultados de los estudios individuales

Los instrumentos de CV relacionada con la piel identificados fueron: el Índice de Calidad de Vida en Dermatología (en inglés *Dermatology Life Quality Index* – DLQI)^(16-20,26,28,31-33,46)^, el Skindex-16^(15,22-25,27,29-30,34-36,38-39,41-45)^, el Skindex-29^(37)^ y el *Padua Skin-Related Quality of Life* (PSRQ)^(21)^. El Skindex-16 fue reportado en estudios desarrollados en EE. UU. (n = 8; 25%), Bélgica (n = 3; 9,375%), Australia (n = 2; 6,25%), Italia (n = 1; 3,125%), Tailandia (n = 1; 3,125%), Irán (n = 1; 3,125%) y Egipto (n = 1; 3,125%). El DLQI fue reportado en estudios desarrollados en EE. UU. (n = 5; 15,625%), Irán (n = 3; 9,375%), Reino Unido (n = 2; 6,25%) y Brasil (n = 1; 3,125%). Los instrumentos Skindex-29 y PSRQ fueron reportados en un estudio cada uno, realizados en Bélgica y EE. UU., respectivamente.

De esta forma, se observa un predominio del uso del Skindex-16 en los continentes de África, América del Norte, Europa y Oceanía, mientras que el DLQI predomina en América del Sur y Asia (Figura 3). Las revisiones sistemáticas, una de Brasil^(40)^ y otra de Japón^(45)^, no fueron incluidas en la Figura 3 para evitar la contabilización duplicada de estudios primarios.

**Figura 3-:** Frecuencia de los instrumentos de CV relacionada con la piel en pacientes con radiodermatitis por continente

El instrumento DLQI^(48)^ tiene como objetivo cuantificar cuánto una afección cutánea afecta la CV de una persona. Se trata de un cuestionario compuesto por 10 ítems divididos en 6 categorías, siendo estas: síntomas y sentimientos (2 ítems), actividad diaria (2 ítems), ocio (2 ítems), trabajo y estudio (1 ítem), relaciones interpersonales (2 ítems) y tratamiento (1 ítem). Cada ítem permite una puntuación de 0 (nada) a 3 (muchísimo) puntos, pudiendo la suma total variar entre 0 y 30 puntos. Al igual que en otros cuestionarios, es importante analizar el contexto sociocultural y económico de la población, ya que se pueden observar diferentes énfasis en las respuestas a las preguntas.

El Skindex^(49)^ consiste en un instrumento de evaluación de la CV dermatológica, cuyo objetivo es medir el impacto de las afecciones dermatológicas y sus efectos en la CV de los individuos. Además, permite distinguir las diferentes manifestaciones cutáneas –como psoriasis, melasma, úlceras crónicas, vitíligo, rosácea, entre otras– y acompañar su evolución a lo largo del tiempo. Evalúa tres dominios, a saber: funcional, emociones y síntomas.

En esta revisión, se identificaron el Skindex-16^(15,22-25,27,29-30,34-36,38-39,41-45)^ y el Skindex-29^(37)^, los cuales comparten algunas especificidades. En ambos cuestionarios, el ítem es desconsiderado cuando se marca más de una respuesta. Para estos instrumentos, es posible cuantificar cada dominio mediante la puntuación media de las respuestas, utilizando una escala de tres niveles. Es decir, cada dominio tiene una media y se evalúan de forma independiente entre sí.

El Skindex-29^(49)^ está compuesto por 29 ítems divididos en los dominios de emociones (10 ítems), síntomas (7 ítems) y funcional (12 ítems). La asignación de puntuación va de 1 punto para “nunca” a 5 puntos para “todo el tiempo”. La puntuación del Skindex-29 se transforma en una escala lineal que varía de 0 a 100 para cada dominio, donde cuanto mayor es la puntuación, mayor es el efecto sobre la CV del individuo. El puntaje total para la evaluación de la Calidad de Vida puede ser de 0 puntos (nunca), 25 puntos (raramente), 50 puntos (a veces), 75 puntos (frecuentemente) y 100 puntos (todo el tiempo). La puntuación final consiste en el promedio de cada dominio; los puntos de corte para identificar pacientes con CV gravemente afectada son ≥ 52 para el dominio de síntomas, ≥ 39 para el de emociones, ≥ 37 para el funcional y ≥ 44 para el puntaje total.

El Skindex-16^(49)^ contiene 16 ítems, de los cuales 7 pertenecen al dominio de emociones, 4 al de síntomas y 5 al funcional. Este instrumento fue desarrollado a partir del Skindex-29 con el objetivo de ser más conciso, preciso y eficiente. Su construcción se basó en la conservación de los ítems con mejor desempeño del Skindex-29 y en la adición de nuevas preguntas. La escala utilizada es de tipo Likert y la puntuación de los ítems varía de 0 puntos para “nunca molestó” a 6 puntos para “siempre molestó”. El puntaje total de cada ítem también se transforma en una escala lineal de 0 a 100 puntos, y la puntuación de cada dominio del Skindex-16 consiste en el promedio de sus ítems.

Por último, el PSRQ^(50)^ es un cuestionario italiano concebido inicialmente con 63 ítems, con el objetivo de medir tanto el nivel de confort como de malestar relacionado con la piel. Actualmente, el PSRQ está compuesto por 50 ítems, y cada ítem permite una respuesta que varía entre 1 “totalmente en desacuerdo” y 5 “totalmente de acuerdo”. El instrumento presenta 5 dominios: compromiso interpersonal (12 ítems), sentimientos y emociones positivas (17 ítems), sentimientos y emociones negativas (13 ítems), estrés y compromiso físico (8 ítems). Su resultado puede obtenerse mediante la suma total de todos los ítems o por dominio, mediante la suma de la puntuación media obtenida por las respuestas de cada conjunto de ítems que conforman el dominio.

Para todos los instrumentos presentados, cuanto mayor es la puntuación, mayor es el compromiso de la CV relacionada con la piel de los individuos. Las principales características de los instrumentos se presentan en la Figura 4.

**Figura 4-:** Características de los instrumentos de CV relacionada con la piel identificados en los artículos seleccionados (n = 32). Brasília, DF, Brasil, 2024

Instrumento	Validado para el portugués	Número de ítems	Número de categorías	Nombre de las categorías	Puntuación	Puntuación atribuida a cada ítem	Tiempo de respuesta en minutos
Índice de Calidad de Vida en Dermatología (DLQI) ^( 48 , 51 )^	Sí	10	6	1.Síntomas y sentimientos	Máxima: 30 / Mínima:0	0 a 3 puntos por cada ítem	Aproximadamente 2
				2.Actividad diaria		0.Nada	
				3.Ocio		1.Un poco	
				4.Trabajo y estudio		2.Mucho	
				5.Relaciones interpersonales		3.Muchísimo	
				6.Tratamiento			
Skindex-16 ^( 49 , 52 )^	Sí	16	3	1.Emoções	Máxima: 96 / Mínima: 0	0 a 6 puntos por cada ítem	2-3
				2.Actividades diarias		0.Nunca molestó	
				3.Síntomas		6.Siempre molestó	
Skindex-29 ^( 49 , 53 )^	Sí	29	3	1.Emociones	Máxima: todo el tiempo/ Mínima: nunca	1.Nunca	Aproximadamente 5
				2.Actividades diarias		2.Raramente	
				3.Síntomas		3.A veces	
						4.A menudo	
						5.Todo el tiempo	
*Padua Skin-Related QoL Questionnair* ^( 50 )^	No	50	5	1.Compromiso interpersonal 2.Sentimientos y emociones positivas 3.Sentimientos y emociones negativas 4.Estrés 5.Compromiso físico	Máxima: 250/ Mínima: 50	1 a 5 puntos por cada ítem	-
						1.Totalmente en desacuerdo
						5. Totalmente de acuerdo

El Skindex-16 y el DLQI fueron los instrumentos más utilizados para evaluar la CV relacionada con la radiodermatitis (Figura 5 ). En lo que respecta al tipo de cáncer de los participantes de los estudios incluidos, se verifica que la CV fue evaluada mayoritariamente en individuos con cáncer de mama (16 estudios utilizaron el Skindex-16^(15,22-25,27,30,34-36,39,41-45)^ y 10 estudios utilizaron el DLQI^(16-20,26,28,31,33,46)^), seguidos por estudios con pacientes con cáncer de cabeza y cuello [5 estudios utilizaron el Skindex-16^(23-24,30,38,43)^ y 2 utilizaron el DLQI^(32,46)^]. El PSRQ^(21)^ fue utilizado en un estudio con pacientes con cáncer de mama, así como el Skindex-29^(37)^.

**Figura 5 –:** Frecuencia de los tipos de cáncer presentados por los pacientes en los estudios que utilizaron el Skindex-16 y el DLQI

## Discusión

En esta revisión de alcance se identificaron cuatro instrumentos utilizados en pacientes con cáncer para evaluar la calidad de vida relacionada con la piel, a saber: DLQI, Skindex-16, Skindex-29 y PSRQ. Se trata de la primera revisión de alcance que mapea instrumentos de CV utilizados para la evaluación de la piel en pacientes con cáncer que fueron sometidos a radioterapia y desarrollaron radiodermatitis.

Las reacciones dermatológicas suelen afectar la autoimagen de los individuos y pueden ocasionar baja autoestima, aislamiento social, ansiedad y depresión^(54-55)^. Además, existen repercusiones físicas, como dolor, prurito y lesiones, que imposibilitan la realización de actividades cotidianas^(56)^. Este contexto afecta la CV de los individuos, ya que provoca alteraciones en los aspectos funcional, emocional, cultural y psicosocial^(56-58)^, influyendo significativamente en el bienestar de las personas. Asimismo, la evaluación de la CV favorece la valoración de los resultados terapéuticos desde la perspectiva del paciente, el seguimiento de la evolución de signos y síntomas, y la elaboración de conductas centradas en disminuir los impactos negativos sobre el paciente^(40,55,57-58)^.

En lo que respecta a la radiodermatitis, la medición de la CV específica de la piel es fundamental en el proceso evaluativo sobre cuánto esta radiotoxicidad afecta la CV del individuo. En un estudio brasileño con 60 mujeres, se observó una incidencia del 100% de irritación cutánea derivada de la radiodermatitis y, mediante el DLQI, fue posible evidenciar su impacto principalmente en el dominio del ocio^(54)^.

Se observó que, a medida que avanzaba el tratamiento y la radiodermatitis surgía o se agravaba, hubo repercusiones negativas en el bienestar de estas mujeres y, en consecuencia, en su CV. Así, la falta de datos sobre los impactos de la radiodermatitis en la CV de los pacientes impide dimensionar cuánto esta condición puede perjudicar, por ejemplo, la autoestima y la autoimagen. Al evaluar tales datos, se posibilita desarrollar un cuidado integral e individualizado de acuerdo con las demandas de cada paciente^(54)^.

Los pacientes con cáncer de mama fueron los más evaluados en cuanto al impacto de la radiodermatitis en la CV^(15-28,30-31,33-37,39,41-46)^. Se considera esencial que estos instrumentos sean evaluados para su aplicación en todos los pacientes con cáncer sometidos a radioterapia, partiendo del supuesto de que desarrollarán algún grado de radiodermatitis durante el tratamiento, independientemente del sitio irradiado. Esto permite observar los efectos de esta reacción adversa sobre la calidad de vida de los pacientes y, así, planificar un cuidado eficiente.

No hay consenso sobre qué instrumento utilizar para evaluar cuánto la toxicidad cutánea derivada de la radioterapia impacta la CV^(54)^, pero es fundamental que las escalas/instrumentos/cuestionarios sean capaces de evaluar aspectos físicos, emocionales y funcionales. El Skindex-16 evalúa los aspectos mentales y emocionales de manera más eficiente que el DLQI. En contrapartida, el DLQI tiene la capacidad de evaluar cuestiones como el funcionamiento diario, la vestimenta, la realización de deportes, y las dificultades encontradas durante el tratamiento, aspectos que no son evaluados por el Skindex-16^(57)^.

Estudios afirman^(25,57)^ que el Skindex-16 ofrece más detalles sobre los síntomas cutáneos que otros instrumentos como el DLQI. El Skindex-16 es más recomendable para individuos con alteraciones leves en la CV, dado que el DLQI es insensible para perjuicios menores en la CV^(54)^. Además, la percepción de los pacientes puede considerarse un factor importante para el control de signos y síntomas a lo largo del tiempo, ya que proporciona datos que pueden no ser percibidos por el profesional de salud^(41)^. Por lo tanto, el Skindex-16 ha sido el instrumento más utilizado para evaluar la CV relacionada con la piel en pacientes con radiodermatitis.

Otro punto importante que debe considerarse al aplicar un instrumento es su validación, ya que esta adapta el cuestionario al público objetivo – en este caso, individuos de la población brasileña con radiodermatitis. Estas herramientas, en su mayoría, son elaboradas de acuerdo con el contexto del país en el que fueron desarrolladas, considerando su entorno social, económico y cultural. Por ello, al ser utilizadas en otros países, es importante que haya una adaptación lingüística, cultural y social que considere el nuevo contexto de evaluación. La validación es también una alternativa más viable que la elaboración de un nuevo instrumento, ya que genera menos costos y estandariza la información compartida por la comunidad científica, facilitando la comprensión y la uniformidad de los resultados^(59)^.

De esta manera, para contar con un instrumento de evaluación de la CV relacionada con la piel, es fundamental considerar la adecuación del lenguaje al público objetivo. También es importante verificar la confiabilidad y la amplitud del instrumento, teniendo en cuenta no solo los aspectos físicos, sino también los psicosociales y culturales^(59)^, ya que la percepción de un individuo sobre determinado factor puede variar según el país de origen, la cultura, las creencias, entre otros factores^(53)^. Estas herramientas proporcionan la perspectiva de los pacientes sobre cómo las condiciones clínicas dermatológicas afectan su vida cotidiana.

Por último, debe considerarse la relevancia del uso de instrumentos que midan la CV dermatológica de los pacientes en la práctica clínica del equipo de enfermería. Son esenciales para promover la seguridad del paciente, ya que reflejan su propia perspectiva sobre el proceso de salud-enfermedad y, en consecuencia, permiten una comunicación eficaz no solo entre el equipo, sino también con el paciente, con el fin de identificar y priorizar cuestiones que necesitan ser abordadas, pero que no han sido identificadas por los profesionales.

Este escenario hace que el paciente sea agente de su propio cuidado y construya estrategias junto con los enfermeros para mejorar los aspectos de su CV afectados, por ejemplo, por la radiodermatitis. El registro de estos instrumentos también puede ayudar al enfermero no solo en la construcción de protocolos institucionales –ya que se pueden observar patrones comunes entre diversos pacientes–, sino también en la promoción de la salud –acciones sanitarias eficaces– y en una posible profilaxis para ciertas problemáticas que afectan la CV.

La presente revisión permite un mayor conocimiento sobre los instrumentos y cuestionarios que evalúan la CV relacionada con la piel. A partir de ello, contribuye en la toma de decisiones sobre cuáles son los instrumentos más adecuados para la evaluación de la CV relacionada con la piel en futuros estudios, además de orientar la práctica clínica y el establecimiento de protocolos en instituciones de salud. Considerando los impactos que la radiodermatitis puede ocasionar en la CV de los pacientes sometidos a radioterapia, este conocimiento es fundamental para brindar una atención calificada que abarque todos los aspectos del cuidado centrado en el paciente.

Entre las limitaciones de esta revisión, puede mencionarse el número limitado de producciones científicas desarrolladas sobre la temática, lo que resultó en una muestra reducida para la síntesis de los datos. Otro limitante fue que gran parte de los estudios presentaron la medición de la CV como desenlace secundario, sin aportar datos más sólidos sobre la temática. Además, estas limitaciones dificultan la formación de un consenso sobre el instrumento más adecuado para medir la CV en la práctica clínica de enfermeros que asisten a pacientes con radiodermatitis. Esto complica un análisis más detallado de los hallazgos clínicos del enfermero y, en consecuencia, impide la construcción de protocolos y cuidados más eficientes.

## Conclusión

Se identificaron diferentes instrumentos utilizados para evaluar la CV relacionada con la piel en pacientes con radiodermatitis, destacándose el Skindex-16 y el DLQI. La diversidad de herramientas apunta a la ausencia de directrices consolidadas sobre la elección del mejor instrumento a ser implementado en este contexto clínico. Por lo tanto, se necesitan más estudios para evaluar el uso de instrumentos en pacientes con radiodermatitis, con el objetivo de determinar cuál es el más adecuado para este fin, la periodicidad de su aplicación y cómo los profesionales de la salud pueden actuar para mejorar los índices y favorecer una mejora en la CV de los pacientes.

## Data Availability

El conjunto de datos de este artículo está disponible en la página de la RLAE en el repositorio SciELO Data, en el enlace https://doi.org/10.48331/SCIELODATA.UBXFJ0.
